# Influenza in Babies

**Published:** 1908-03-14

**Authors:** 


### Diseases of Children.

### INFLUENZA IN BABIES.

It is generally asserted that babies are not liable to influenza, and it certainly appears a justifiable assumption that they are less susceptible than older children and adults, and that as a rule they are attacked less severely. The smaller liability mainly depends on the less frequent exposure to infection, and the lack of severity to the fact that babies are more carefully protected when ill and are kept warm and quiet during even trivial ailments.

Probably many of the cases of partial lung consolidation, at present unduly frequent among babies, are the result of broncho-pneumonic conditions secondary to influenza. The differentiation of these cases from the commoner forms of broncho-pneumonia is impossible, but suggestive features are the presence of influenza in the house, a history of exposure to infection, a febrile attack of a day or two's duration, followed after an interval by bronchitis and pneumonia, and the presence of an amount of depression in undue proportion to the lung affection, an excessively rapid pulse and respiration rate, and cardiac weakness.

Occasionally influenza produces severe and serious symptoms in babies, and diagnosis may be impossible until some days have elapsed, and, perhaps, then only by a process of exclusion. A case of this type may be mentioned. An eight months old girl, always previously healthy, was feverish and ailing one night, after having been out in a bitterly cold east wind. Next night she had convulsions, and was more feverish. In another three days she was apparently well. On the seventh day she had been free from fever for two days, but at mid-day she became convulsed, and her temperature rose to 103° F. A few hours later the temperature had fallen to 99.2° F., and nothing abnormal could be discovered. During the next twenty-four hours she had three mild fits, and at the end of this time her temperature was 104.2° F., pulse 168, respirations 42. For another six days the temperature ran an irregular course, sometimes high, at others almost normal. On examination her pulse-rate was still 158, respirations 48, and she seemed brighter, though she took food badly. From this day onward she made a steady recovery. Now here was an illness which might at the onset have been due to innumerable causes. On the three occasions the child was seen in consultation, a most careful and thorough examination was made of all the organs of the body, and every one was found apparently normal. The urine was normal. Yet the fever, pulse, and respiration, the convulsions and the prostration indicated a profound toxæmia. Such a toxæmia might have been produced by the pneumococcus, the tubercle bacillus or by influenza, as well as other organisms. The fact that the acute illness followed a mild febrile attack, without any local cause for the condition, led to the diagnosis of influenza. In the early stages the important diseases to bear in mind were the various types of meningitis, acute otitis, throat affections, pyelitis, typhoid and paratyphoid infections, and even general tuberculosis. The case illustrated a valuable hint on prognosis, namely that the outlook of high fever in babies and children is generally favourable in those cases in which no definite cause for the fever can be found after careful and repeated examination. Treatment in a patient of the type mentioned must be directed on general principles. It is imperative to prevent, if possible, the convulsions, and both bromides and chloral may be given freely. Reduce the diet so that it is liquid and easily digestible, and keep the bowels open. Later on the cardiac weakness will require attention, and usually nux vomica is the best drug for this purpose.

Tachycardia sometimes arises in babies after influenza. It occurred in a baby eight months old, as a sequel of a feverish attack lasting seven days, the temperature ranging from 100° F. to 104° F. without any definite symptoms. In addition to the tachycardia the child had attacks of collapse and lividity. The apex of the heart was an inch outside the nipple line. In a three months old baby the tachycardia followed what was apparently an ordinary influenzal cold of the old-fashioned type. Attacks of collapse and lividity, or of syncope, may occur after simple acute coryza and in pertussis. Sometimes, therefore, these heart affections are due to the toxin of true influenza or of acute coryza, etc., or depend on cardiac dilatation, the result of the action of the poison or of strain, by coughing, on the myocardium. Caffein, nux vomica, bromides, belladonna, and stimulants during the syncopal attacks are needed. It may be necessary to give strychnia, ether, ammonia, or brandy subcutaneously, for such patients may die unless actively treated.

A third form of influenza in babies, liable to give trouble in diagnosis and prognosis, is the abdominal type. It is characterised by abdominal pain, retraction or distension of the abdomen, constipation, vomiting, restlessness, and occasional crying. Sometimes it is preceded by diarrhœa. Such cases are very suggestive of the onset of tuberculous meningitis, for there may be little or no fever at the time the child is seen. In a few instances appendicitis is suggested.

The treatment of influenza in babies is comparatively simple, for warmth, rest, and careful feeding are usually the only essential measures. Brandy may be required. A careful watch must be kept on the heart and lungs for the development of complications. The bowels should be kept open, and simple tonics, such as the syrup of the glycerophosphate or phosphate of iron, be given when the fever has subsided. Great care is essential in guarding such children against chill during convalescence, and in ensuring a liberal supply of fresh air in the sick-room, by night and by day, without exposing the child to a direct draught. Antipyretics may be necessary in early stages, but all drugs liable to upset the stomach should be used very cautiously. Bromides are most valuable for the restlessness and irritability.

